# Graduating Exercises of the Atlanta School of Medicine

**Published:** 1907-06

**Authors:** 


### GRADUATING EXERCISES OF THE ATLANTA SCHOOL OF MEDICINE.

Twenty men were graduated from the Atlanta School of Medicine April 23rd. The exercises were held in the Grand Opera House, Dr. W. W. Landrum opened with prayer, then Dr. George H. Noble, Dean, read his report. The diplomas were delivered by Bishop C. K. Nelson and the annual oration by Dr. Jas. W. Lee.

Certificates were awarded to the six highest in proficiency as follows: Robt. F. Cary, John W. Chambliss, Oscar F. Collins, Edward D. Crawford, James A. Fort and Clyde A. Stevenson.

Those who received degrees were:

J. L. Adams, Georgia; H. M. Baker, Texas; D. R. Bridges, Georgia; C. R. Bullock, Georgia; E. F. Carter, Georgia; R. F. Cary, Georgia; J. W. Chambliss, Georgia; O. F. Collum, Georgia; E. D. Crawford, Georgia; B. V. Elmore, Georgia; J. A. Fort, Georgia; J. M. Harris, Oklahoma; W. L. Harris, Oklahoma; W. L. Hogue, Georgia; A. B. Jones, Georgia; D. A. Powell, North Carolina; H. L. Sams, Georgia; B. K. Simmons, Alabama; C. A. Stevenson, Georgia; J. W. Thomas, Georgia; D. B. Williams, Georgia.

The Dean's report showed that this institution was in an excellent condition, having had enrolled 221 men during the session of 1906 and 1907 who received the advantages of the new buildings and college hospital which, with the grounds and equipment have cost between $80,000 and $90,000. The abundance of clinical material and the use of the hospital which is operated exclusively by the college, enabled the faculty to devote more attention to this part of the course than has formerly been given.

